# Effects of soybean hull inclusion on dietary energy value and nitrogen balance in growing gilts

**DOI:** 10.1093/tas/txag016

**Published:** 2026-02-14

**Authors:** Mariah L Mayer, Dalton C Humphrey, Laura L Greiner

**Affiliations:** Department of Animal Science, Iowa State University, Ames, IA, 50011, United States; Department of Animal Science, Iowa State University, Ames, IA, 50011, United States; Department of Animal Science, Iowa State University, Ames, IA, 50011, United States

**Keywords:** energy, gilts, nitrogen, soybean hulls, swine

## Abstract

The objective of this study was to determine the effect of increasing dietary soybean hull inclusion on diet digestibility, energy content, and nitrogen (N) balance in growing gilts. A 21-day study was conducted with two groups of 24 gilts with a starting body weight of 45.5 kg ±2.2 kg housed in individual metabolism stalls. Gilts were fed one of four dietary treatments containing either 0%, 10%, 20%, or 40% soybean hulls for a total of 12 gilts per treatment. The four diets were formulated to equal a standardized ileal digestibility (SID) lysine: metabolizable energy (Lys: ME) of 3.41. During the 21 days, gilts were evaluated over three collection periods. Each period consisted of four days of total fecal and urine collection. Gilts remained on their respective diets for the duration of the study. Urine, fecal, feed, and soybean hull samples were analyzed for N and gross energy concentration. Data were analyzed using SAS 9.4 using the GLIMMIX procedure, and gilt was the experimental unit. Results were considered significant if *P ≤* 0.05. Feed intake was not different as soybean hull inclusion increased but increased across collection periods (*P <* 0.001). Apparent total tract digestibility (ATTD) of crude protein decreased linearly as soybean hull inclusion increased (*P ≤* 0.001). Fecal N excretion increased as soybean hull inclusion increased (*P ≤* 0.001) and over the three collection periods (*P <*0.001). Total urine N excretion increased over time and soybean hull inclusion caused a linear reduction in urinary N excretion (*P ≤* 0.001). Nitrogen retention (g/d) was reduced by greater soybean hull inclusion (*P = *0.028). Metabolizable energy (ME) decreased as soybean hulls inclusion increased in the diets (*P ≤* 0.001). In conclusion, increasing soybean hulls up to 40% in the diet increased fecal N excretion and decreases urine N excretion and the ATTD of N. Additionally, the increase in soybean hulls in the diet decreased the ME value, while ME values increased as the time on the diet increased.

## Teaser

Nutrient digestibility decreased and fecal nitrogen excretion increased as dietary soybean hull inclusion increased from 0 to 40%. The feeding of increased levels of soybean hulls resulted in reduced metabolizable energy content.

## Introduction

Soybean hulls are removed from soybeans in order to manufacture soybean oil and soybean meal (Stein et al. 2008). The hull that is removed from the soybean contains both insoluble and soluble fiber at 69.5% and 13.3% respectively, or an ADF value of 43% and an NDF value of 67%, making it a substantial source of dietary fiber (Dust et al. 2004). Therefore, soybean hulls may be a viable fibrous ingredient for diluting the energy concentrations of the diet, thereby maintaining growth through promoting feed intake, gut fill, and satiety (Wenk 2001; Kerr and Shurson 2013; Souza da Silva et al. 2013).

Unlike ruminants, monogastric animals do not have exogenous enzymes necessary in the gut for the breakdown of fiber for proper digestion (Lewis and Southern 2000; Stein et al. 2008). However, fiber digestion does occur in the monogastric animal within the large intestine where microbial populations ferment the fiber (Jha et al. 2019; Hu et al. 2023). The fermentability of fiber is dependent on its solubility, viscosity, degree of lignification, water-holding capacity, and physical structure (Gonzalez-Ortiz et al. 2019). Fiber concentration in swine diets can have both positive and negative impacts on animal performance. Select fiber sources can provide merit such as improved gut health and reduced constipation; however, high fiber content can increase gut fill and reduce animal feed intake reducing growth rates (Agyekum and Nyachoti 2017). Dietary fiber alters a pig’s digestive tract by enlarging its size, volume, and morphology structures when fed over a long-term period (Lewis and Southern 2000; Jha and Berrocoso 2015), allowing adaptation to a fibrous diet through increased fermentation capabilities (Kerr and Shurson 2013). Insoluble fiber increases bulking capacity which can lead to reduce growth rates while increasing passage rate (Freire et al. 2000; Jha and Berrocoso 2015), but it can also stimulate the growth of intestinal villi, and positively affects gut motility (Patience and Petry 2019).

However, the feeding of high levels of fiber, such as soybean hulls, can result in reduced digestibility of protein and carbohydrates (Lancheros et al. 2022). Recent data suggest that feeding of soybean hulls to growing pigs resulted in a metabolizable energy value estimates ranging from 1,893 to 3,130 kcal/kg of feed as-fed (Rodriguez et al. 2020; Kim et al. 2025). The significant variation in soybean hull energy values can be associated with the apparent total tract digestibility of the gross energy (Kim et al. 2025); however, further research is warranted to fully understand the energy values associated with soybean hull. The hypothesis of this study was that nutrient digestibility, nitrogen retention, and metabolizable energy would decrease with increased inclusion of soybean hulls. Therefore, this study aimed to estimate the metabolizable energy value of the diets and assess the impact of increasing dietary levels of soybean hulls on nitrogen digestibility in growing gilts.

## Materials and methods

All experimental protocols adhered to guidelines for the ethical and humane use of animals for research according to the Guide for the Care and Use of Agricultural Animals in Research and Teaching (FASS 2010) and were approved by the Institutional Animal Care and Use Committee at Iowa State University (IACUC 23–164).

### Animals, housing, and experimental design

This experiment was conducted from November 2023 to March 2024 at Iowa State University Swine Nutrition Farm (Ames, IA, USA), utilizing two groups of 24 growing gilts (PIC 337* × *1050, Genus, Hendersonville, TN, USA) with an initial body weight (BW) of 46 ± 3.4 kg. At the start of the experiment, pigs were weighed and placed into metabolism stalls (0.7 m x 1.5 m) that contained a slatted floor, feeder, and water nipple. The stalls were housed in the same temperature-controlled room, maintained at a temperature of approximately 21°C.

### Dietary treatments

Upon entry into metabolism stalls, pigs were randomly allocated to one of four dietary treatments (*n* = 6/group). The dietary treatments contained increasing levels of loose soybean hulls (0, 10, 20, 40%; Table 1) sourced from a commercial supplier (ADM, Des Moines, IA, USA). The nutrient values of corn, soybean meal, and soybean hulls used in diet formulation were based on NRC (2012) values. Additionally, diets were formulated to contain a standardized ileal digestible (SID) lysine to metabolizable energy ratio (Lys: ME) of 3.41, and all other nutrients met or exceeded the NRC (2012) requirement estimates. All pigs were allowed ad libitum access to with feed and water for the duration of the experiment.

**Table 1:** Ingredient and nutrient composition of experimental diets, as-fed basis.

	Soyhull inclusion			
**Ingredient**	0%	10%	20%	40%
**Soybean hulls**	0.00	10.00	20.00	40.00
**Corn**	67.85	60.22	52.56	36.80
**Soybean meal**	29.81	27.68	25.55	20.62
**Sodium chloride**	0.50	0.50	0.50	0.50
**Calcium carbonate**	0.49	0.31	0.13	0.00
**Monocalcium phosphate**	0.47	0.47	0.48	1.38
**L-lysine HCl**	0.28	0.25	0.22	0.17
**DL-methionine**	0.12	0.11	0.11	0.10
**L-threonine**	0.10	0.09	0.08	0.07
**L-valine**	0.02	0.01	0.01	0.00
**Phytase^a^**	0.01	0.01	0.01	0.01
**VTM premix^b^**	0.35	0.35	0.35	0.35
**Total**	100.00	100.00	100.00	100.00
**Calculated composition**				
**Dry matter, %**	87	87	88	88
**Crude protein, %**	19	19	18	17
**SID Lys, %**	1.10	1.05	1.01	0.90
**SID Ile: Lys**	0.61	0.61	0.62	0.62
**SID Arg: Lys**	1.06	1.02	0.97	0.86
**SID His: Lys**	0.39	0.37	0.35	0.31
**SID Leu: Lys**	0.42	0.39	0.36	0.28
**SID Thr: Lys**	0.61	0.61	0.61	0.61
**SID Trp: Lys**	0.18	0.18	0.18	0.18
**SID Val: Lys**	0.67	0.67	0.67	0.67
**SID Phe: Lys**	0.74	0.75	0.75	0.76
**SID Met: Lys**	0.34	0.34	0.34	0.34
**SID Met + Cys: Lys**	0.56	0.56	0.56	0.56
**ME, Mcal/kg**	3.23	3.10	2.96	2.65
**SID Lys: ME, g/Mcal**	3.41	3.41	3.41	3.41
**Analyzed composition**				
**Dry matter, %**	85.81	86.28	87.05	88.02
**Ash, %**	3.85	3.92	4.00	4.67
**Gross energy, kcal/kg**	3,839	3,880	3,896	3,885
**Crude protein, %**	18.32	18.00	17.31	16.40

a Quantum Blue 5G (AB Vista, Marlborough, U.K.); provided 500 FTU/kg, assuming 0.15% available phosphorus and 0.165% calcium release.

b Vitamin trace mineral premix; provided 9,011 IU vitamin A, 2,254 IU Vitamin D, 60 IU vitamin E, 3.8 mg vitamin K, 7.1 mg riboflavin, 39.9 mg niacin, 2.2 mg thiamin, 1.7 mg folic acid, 0.2 mg biotin, 20 mg Cu (copper sulfate and basic copper chloride), 0.7 mg I (ethylenediamine dihydroiodide), 100 mg Fe (ferrous sulfate), 50.1 mg Mn (manganous oxide), 0.3 mg Se (sodium selenite and selenomethionine hydroxy analogue), and 120 mg Zn (zinc sulfate) per kg of the diet.

### Sample collection

Diet samples for each treatment were collected at the time of mixing each batch of feed and stored at -20°C until subsequent analysis. The 21-day experiment consisted of three 96 h collection periods, which began at 0700 h on days 3, 10, and 17. The pigs were allowed three days of acclimation to diets and housing in metabolism stalls prior to the first collection period. Total quantities of urine and feces were collected twice daily during each collection period at 0700 h and 1600 h and immediately stored at -20°C. Urine was collected into stainless steel buckets containing 25 mL of 6 N HCl to prevent bacterial growth and prevent nitrogen volatilization (Kim et al. 2023).

Feed left (orts) at the end of each collection period was collected from the feeders and stored at -20°C. Feed intake was determined within each collection period by accounting for the weight of dried feed refusals. The pigs were weighed at the beginning of the experiment and at the end of each collection period to calculate average daily gain (ADG).

### Sample analysis

Soybean hull samples were sent to Ward Laboratories (Kearney, NE, USA) for analysis of moisture, dry matter (DM; Shreve et al. 2006), crude protein (CP; AOAC method 990.3), ADF (ANKOM 2020a; method 14.2020), NDF (ANKOM 2020b; method 15.2020), crude fat (AOCS 2024; Am 5-04), ash (AOAC 942.05), total starch (AOAC 996.11), and gross energy (GE; NRC 2012). Soybean hull samples were analyzed for micron size (μm) using a 7-layer sieve. Soybean hull samples were also tested for trypsin inhibitor units (TIU) at University of Missouri Agricultural Experiment Station Laboratories (Columbia, MO, USA; AACC 22–40, 2006).

Orts were oven-dried at 75°C to a constant weight and calculated back into DM intake. Total fecal collections from each period were also oven-dried at 75°C to a constant weight, then ground and subsampled. Dried feces and diet samples were ground through a 1 mm screen using a Wiley Mill (Variable Speed Digital ED-5 Wiley Mill; Thomas Scientific, Swedesboro, NJ, USA). Urine samples were thawed, homogenized, subsampled, and filtered through glass wool twice, then stored in plastic screw-top containers at -20°C until further analysis.

Diet, ingredient, and dried fecal samples were analyzed in duplicate for DM (method 930.15; AOAC 2007) and ash. Dry matter and ash were calculated by determining the mass difference after oven drying for 24 hours at 100°C and 12 hours at 600°C, respectively. Diet, fecal, and urine samples were analyzed in duplicate for nitrogen (N) by the Dumas combustion method using an automatic N analyzer (method 990.03 AOAC 2007; TruMac N; LECO Corporation, St. Joseph, MI, USA). Crude protein was calculated as *N × *6.25.

Diet, fecal, and urine samples were analyzed in triplicate for GE using an isoperibolic bomb calorimeter (Model 6200; Parr Instrument Company, Moline, IL, USA). Benzoic acid (6,318 kcal/kg) was used for standard calibration and was determined to contain 6318 ± 18 kcal/kg. To determine the GE of urine, 3 mL of urine was added to 0.50 g of cellulose (Acros Organics, Geel, Belgium) and dried for 72 h at 50°C. Dried urine plus cellulose samples were analyzed in triplicate for GE. Urine GE was calculated from the difference in energy determined in cellulose alone and the samples with both urine and cellulose. The water holding capacity (WHC) of dietary fiber was measured by weighing 0.25 to 0.50 g of the sample into 15 mL conical centrifuge tubes, adding 10 mL of distilled water, and soaking at room temperature for 24 hours (Robertson et al. 2000). The samples were centrifuged at 6000 × g for 15 minutes. The volume of water retained was measured and recorded. Water holding capacity was calculated as the volume of retained water per gram of dry matter.

### Calculations and statistical analysis

Digestible energy was calculated by subtracting fecal energy from GE intake. Metabolizable energy was calculated by subtracting urine and fecal energy from GE intake. Digestibility and nutrient balance values were calculated using the following equations previously reported in Humphrey et al. (2022):


Apparent total tract digestibility (ATTD)=[(nutrient in feed×feed intake) −(nutrient infeces×fecal output)] / (nutrient in feed×feed intake)

Total nutrient excretion=fecal nutrient excretion       +urine nutrient excretion

Nutrient retention=nutrient intake−total nutrient excretion

Retention (% intake)=nutrient retention/nutrient intake

Retention (% digestible)=nutrient retention          /(nutrient intake×ATTD coefficient)

Data were analyzed as repeated measures according to the following statistical model:


$$y_{\mathrm{ijkl}}=\mu +T_{i}+P_{j}+{\left(T\times P\right)}_{ij}+B_{k}+e_{\mathrm{ijkl}}$$

Where $y_{\mathrm{ijkl}}$ is the observed value for the $l^{th}$ gilt fed the $i^{th}$ level of soybean hull inclusion during the $j^{th}$ collection period from the $k^{th}$ group; μ is the overall mean; $T_{i}$ is the fixed effect of the $i^{th}$ level of soybean hull inclusion (i = 1 to 4); $P_{j}$ is the fixed effect of the $j^{th}$ collection period (j = 1 to 3); ${\left(T\times P\right)}_{ij}$ is the two-factor interaction between soyhull inclusion and collection period; $B_{k}$ is the fixed effect of the $k^{th}$ group; $e_{\mathrm{ijkl}}$ is the random error associated with $y_{\mathrm{ijkl}}$. Repeated observations across collection periods within gilt were modeled using candidate covariance structures, including compound symmetry (CS), CS with heterogenous variance, first-order autoregressive [AR(1)], and AR(1) with heterogenous variance. Covariance matrices for each response variable were selected as best fit based on Akaike information criterion (AIC).

All models were implemented in SAS 9.4 (SAS Institute, Cary, NC, USA) using the GLIMMIX procedure. The normality and homoscedasticity of the Studentized residuals for all models were verified using the UNIVARIATE procedure. Studentized residuals greater than approximately three standard deviations from the mean were considered statistical outliers and excluded from the analysis. Results are reported as least squares means, and means separation was conducted using the PDIFF option with Tukey adjustment for multiplicity. Additionally, orthogonal polynomial contrasts were constructed to test linear and quadratic effects of soyhull inclusion. The individual pig was considered the experimental unit, and results were considered significant if *P ≤* 0.05.

## Results

### Diet analysis

The analyzed CP concentration of the diets was slightly lower than that of the calculated; however, the CP concentration did decline as soybean hull inclusion increased (Table 1). Analyzed ash content was higher as the soybean hulls level increased in the diets. Soybean hull samples (Table 2) had an ADF value of 45.41% and an NDF value of 52.90%. The water holding capacity of soybean hulls samples ranged from 7 to 8%. Micron size of the soybean hulls used in the current study averaged 690 ± 73.15 μm and included a trypsin inhibitor concentration 5.31 TIU/mg (Table 2).

**Table 2:** Analyzed nutrient composition of soybean hulls.

Item	Composition, as-fed
**Dry matter, %**	91.58
**Gross energy, kcal/kg**	3,835
**Ash, %**	3.97
**Crude protein, %**	9.79
**ADF, %**	45.41
**aNDF^a^, %**	52.90
**Crude fat, %**	1.20
**Starch, %**	0.27
**Trypsin inhibitor, TIU/mg**	5.31
**Particle size, μm**	690

a Amylase-treated NDF.

### Growth performance

Pigs began the trial at an initial average BW of 45.5 ± 2.2 kg with similar average weights among dietary treatments (*P = *0.24; Table 3). Pigs were weighed at seven-day intervals with a final weight recorded on day 21. As soybean hull inclusion increased, body weight decreased during the three-week trial period (*P* = 0.022) due to decreased average daily gain (*P* = 0.020). Average daily feed intake was not affected by dietary treatments (*P = *0.643) but increased throughout the collection period (*P ≤* 0.001).

**Table 3:** Effect of soybean hull inclusion and collection period on growing gilt body weight, average daily gain, feed intake, fecal and urine output, and ATTD of diet dry matter, ash, organic matter, and crude protein.

	Soybean hull inclusion					*P*-value^a^			Period				*P*-value	
Item	0%	10%	20%	40%	SEM	Trt	LN	QD	1	2	3	SEM	Period	Trt* × *Period
**Body weight, kg^b^**						0.237							<0.001	0.162
** d 0**	45.58	45.55	45.43	45.57	0.722		0.981	0.884	–	–	–	–		
** d 7**	51.37	51.19	50.25	49.27	0.885		0.057	0.936	–	–	–	–		
** d 14**	58.62	58.24	56.73	55.77	0.972		0.021	0.840	–	–	–	–		
** d 21**	67.07	65.95	64.57	64.01	0.988		0.022	0.437	–	–	–	–		
**Average daily gain, kg/d**	1.02	0.97	0.98	0.88	0.042	0.113	0.020	0.805	0.71^z^	1.00^y^	1.17^x^	0.036	<0.001	0.287
**Feed intake, g/d as-fed**	2,155	2,158	2,021	2,104	88.8	0.643	0.555	0.451	1,831^z^	2,141^y^	2,356^x^	61.6	<0.001	0.270
**Dry fecal output, g/d**	172^a^	219^ab^	258^b^	408^c^	21.6	<0.001	<0.001	0.248	247^x^	264^xy^	281^y^	13.2	0.049	0.797
**Urine output, g/d**	2,247^b^	1,877^ab^	1,576^a^	1,511^a^	161.4	0.007	0.002	0.113	1,481^x^	1,936^y^	1,991^y^	103.4	<0.001	0.277
**Diet ATTD, %**														
** Dry matter**	90.76^a^	88.34^a^	85.21^b^	77.77^c^	0.722	<0.001	<0.001	0.216	84.13^y^	85.94^x^	86.49^x^	0.537	0.002	0.201
** Ash**	75.00^a^	73.00^ab^	69.30^b^	62.06^c^	1.226	<0.001	<0.001	0.457	67.02^y^	71.58^x^	70.92^x^	1.170	0.001	0.521
** Organic matter**	91.50^a^	89.08^a^	85.95^b^	78.65^c^	0.712	<0.001	<0.001	0.244	85.00^y^	86.65^x^	87.23^x^	0.526	0.003	0.212
** Crude protein**	89.51^a^	85.61^b^	82.01^c^	74.60^d^	0.726	<0.001	<0.001	0.907	81.87^y^	83.33^x^	83.60^xy^	0.617	0.024	0.386

a-d Within a row, dietary treatment means without a common superscript differ (*P ≤* 0.05).

x-z Within a row, period means without a common superscript differ (*P ≤* 0.05).

a LN = linear; QD = quadratic.

b Body weight reported as Trt* × *Period least squares means; Linear and quadratic contrasts were tested within period.

### Apparent total tract digestibility

Ash ATTD was influenced by dietary treatment (*P ≤* 0.001), with pigs fed the 40% soybean hulls diet having 12.94% reduced ATTD compared to pigs fed the basal diet (0%; Table 3). Ash ATTD was significantly lower (*P <*0.001) in period one compared to periods 2 and 3; however, there was no interaction among dietary treatments and collection periods (*P = *0.201). Dietary treatment negatively affected the ATTD of DM with pigs fed a diet containing 40% soybean hulls having the lowest ATTD when compared to pigs fed the 0% soybean hulls control diet (*P <*0.001; linear). Additionally, ATTD of DM increased over time (*P = *0.002). Crude protein ATTD was linearly reduced with the 40% soybean hulls diet (*P ≤* 0.001). Pigs fed the 40% soybean hulls diet had decreased (*P ≤* 0.001; linear) ATTD of OM compared to the diet with 0% soybean hulls, but there was an increase in ATTD over time (*P = *0.003).

### Fecal and urine output

Urinary output was higher (*P = *0.007) from pigs from 0% soybean hulls compared to pigs fed either the 20% or 40% soybean hulls diets (Table 3). Collection period influenced urinary (*P ≤* 0.001) and fecal output (*P = *0.049). Fecal output (g/d) increased linearly with greater soybean hulls inclusion (*P <*0.001) with the pigs fed the 40% soybean hulls diet having the greatest fecal output compared to the other three treatment groups.

### Nitrogen balance

Nitrogen intake per day had no treatment by period interaction (*P = *0.461) but increased over time (*P <*0.001; Table 4). Nitrogen intake per day decreased linearly from 63.17 to 55.23 g/d as soybean hull inclusion increased (*P = *0.013). Fecal N excretion (g/d) was higher for the pigs fed the 40% soybean hulls diet (*P ≤* 0.001; linear) compared to the other three dietary treatments. Fecal N excretion also increased over the collection period of the study (*P = *0.007). Pigs fed the 40% soybean hulls diet had lower urinary N output compared to the 0% soybean hulls treatment group with the 10% and 20% soybean hull inclusion treatment groups being intermediate (*P <*0.001; linear). Total N excretion increased over time (*P ≤* 0.001) but was not affected by dietary treatment (*P = *0.590). Urine to fecal ratio decreased linearly as soybean hull inclusion increased (*P ≤* 0.001) with pigs fed the 40% soybean hulls diet having the lowest ratio compared to the other three treatment groups. There was no difference in urine to fecal ratio by collection period (*P = *0.729). Nitrogen retention (g/d) had no interaction between treatment and period (*P = *0.640) but increased over time (*P <*0.001) with the highest N retention being observed in period 3. Nitrogen retention was linearly reduced as soybean hull inclusion increased (*P = *0.004). Nitrogen retention when based on percent of N intake did not change over time (*P = *0.949), but did decrease linearly as soybean hull inclusion increased (*P = *0.013). Further evaluation of N retention as a percent of digestible N showed no treatment by period interaction (*P = *0.261), but there was a linear increase as soybean hulls inclusion increased (*P <*0.001).

**Table 4:** Effect of soybean hull inclusion and collection period on the nitrogen balance of growing gilts.

	Soybean hull inclusion					*P*-value			Period				*P*-value	
Item	0%	10%	20%	40%	SEM	Trt	LN	QD	1	2	3	SEM	Period	Trt* × *Period
***N* intake, g/d**	63.17	62.01	55.94	55.23	2.526	0.053	0.013	0.455	51.36^z^	59.97^y^	65.93^x^	1.752	<0.001	0.461
***N* excretion, g/d**														
** Fecal**	6.71^a^	9.03^ab^	10.01^b^	13.86^c^	0.684	<0.001	<0.001	0.990	8.97^x^	9.96^xy^	10.78^y^	0.447	0.007	0.681
** Urine**	14.83^d^	12.72^c^	10.32^b^	7.43^a^	0.528	<0.001	<0.001	0.238	9.45^x^	11.42^y^	13.10^z^	0.403	<0.001	0.075
** Total**	21.82	21.55	20.01	21.30	1.023	0.590	0.635	0.303	18.56^x^	21.42^y^	23.52^z^	0.788	<0.001	0.279
**Urinary *N*: Fecal *N***	2.29^d^	1.49^c^	1.09^b^	0.57^a^	0.106	<0.001	<0.001	0.003	1.29	1.35	1.44	0.078	0.379	0.729
***N* retention, g/d**	41.36^a^	40.48^ab^	36.54^ab^	34.01^b^	1.928	0.028	0.004	0.821	32.80^z^	39.15^y^	42.35^x^	1.244	<0.001	0.640
***N* retention, % of *N* intake**	65.50	65.67	64.09	61.13	1.388	0.081	0.013	0.509	63.91	64.28	64.10	1.004	0.949	0.288
***N* retention, % of dig. *N***	73.13^c^	75.91^bc^	79.10^ab^	81.52^a^	1.249	<0.001	<0.001	0.264	77.72	77.75	76.78	1.001	0.486	0.261

a-d Within a row, dietary treatment means without a common superscript differ (*P ≤* 0.05).

x-z Within a row, period means without a common superscript differ (*P ≤* 0.05).

### Energy value

Estimated gross energy intake was the same among dietary treatments (*P = *0.871) but increased over time (*P ≤* 0.001; Table 5). There was a collection period response for digestible energy with the final period demonstrating the greatest DE compared to collection period 1 and 2 (*P = *0.008). Digestible energy was linearly affected by dietary treatment (*P ≤* 0.001) with the 40% soybean hulls diet having the lowest DE (2,974 kcal/kg, as-fed basis) compared to the 0% soybean hulls diet (3,435 kcal/kg, as-fed basis). Metabolizable energy was also linearly influenced by dietary treatment (*P ≤* 0.001) with the 40% soybean hulls diet having the lowest ME (2,892 kcal/kg, as-fed basis) compared to the other three dietary treatments. Energy ratios (DE: GE and ME: GE) were statistically lower when pigs were fed 40% soybean hull inclusion compared to the 0% soybean hull diets (*P <*0.001; linear). The ratios were statistically higher from period 1 of the study compared to periods 2 and 3 (*P <*0.01). The ME: DE ratio was higher in the 40% soybean hull inclusion diet compared to the 0% soybean hull diet (*P = *0.005; linear).

**Table 5:** Effect of soybean hull inclusion and collection period on the concentrations of DE and ME in experimental diets.

	Soybean hull inclusion					*P*-value			Period				*P*-value	
Item	0%	10%	20%	40%	SEM	Trt	LN	QD	1	2	3	SEM	Period	Trt* ×*Period
**GE intake, kcal/d**	8,258	8,368	7,990	8,173	336.7	0.871	0.727	0.753	7,092^z^	8,289^y^	9,211^x^	239.5	<0.001	0.136
**Excretion, kcal/d**														
** Fecal**	879^a^	1,080^ab^	1,261^b^	1,900^c^	96.7	<0.001	<0.001	0.291	1,180^x^	1,277^xy^	1,384^y^	60.7	0.009	0.550
** Urine**	256^c^	233^bc^	204^ab^	170^a^	10.4	<0.001	<0.001	0.522	188^x^	212^y^	246^z^	11.7	<0.001	0.687
**Energy content**														
** DE, kcal/kg as-fed**	3,435^a^	3,380^a^	3,266^b^	2,974^c^	29.9	<0.001	<0.001	0.049	3,216^y^	3,283^x^	3,292^x^	20.7	0.008	0.461
** DE, kcal/kg DM**	4,002^a^	3,916^a^	3,752^b^	3,379^c^	34.5	<0.001	<0.001	0.070	3,708^y^	3,784^x^	3,795^x^	23.9	0.009	0.445
** ME, kcal/kg as-fed**	3,315^a^	3,270^ab^	3,165^b^	2,892^c^	30.6	<0.001	<0.001	0.055	3,111^y^	3,180^x^	3,191^x^	21.4	0.007	0.294
** ME, kcal/kg DM**	3,862^a^	3,788^a^	3,636^b^	3,286^c^	35.3	<0.001	<0.001	0.077	3,586^y^	3,665^x^	3,679^x^	24.7	0.007	0.281
**Energy efficiency, %**														
** DE: GE**	89.44^a^	87.15^ab^	83.82^c^	76.55^d^	0.728	<0.001	<0.001	0.245	83.03^y^	84.72^x^	84.96^x^	0.576	0.011	0.527
** ME: DE**	96.51^b^	96.74^ab^	96.83^ab^	97.22^a^	0.174	0.042	0.005	0.987	96.73	96.94	96.80	0.137	0.440	0.679
** ME: GE**	86.33^a^	84.30^a^	81.22^b^	74.39^c^	0.765	<0.001	<0.001	0.255	80.30^y^	82.04^x^	82.33^x^	0.544	0.008	0.299

a-d Within a row, dietary treatment means without a common superscript differ (*P ≤* 0.05).

x-z Within a row, period means without a common superscript differ (*P ≤* 0.05).

## Discussion

In the present study, BW did decrease as soybean hull inclusion increased, which aligns with Miller et al. (2024), who reported a linear decrease in grow-finish pig BW as soybean hulls increased to 22.5% of the diet. However, previous work by Stewart (2007) showed no differences in body weight as soybean hull inclusion increased. Similarly, ADFI did not differ among dietary treatments in the current study, which agree with studies by Stewart (2007) and Miller et al. (2024) in which both papers reported no effect of increasing dietary soybean hulls on ADFI. In contrast, Kornegay (1978) reported an increase in feed intake with increasing soybean hull inclusion (2–24% inclusion) in nursery pigs and Goehring et al. (2019) reported a decrease in feed intake in nursery pigs when as soybean hull inclusion increased from 0 to 20% inclusion. These discrepancies may result from differences in soybean hulls source, inclusion, or composition (Agyekum and Nyachoti 2017). The inclusion level can also have an effect on performance, as a low inclusion level of fiber may not elicit any impacts on growth performance compared to a diet high in fiber. Different soybean hulls sources can vary in production methods which can play a role in micron size. The soybean hulls used in the current study had an average particle size of 690 ± 73.15 μm and contained 5.31 TIU/mg. Goehring et al. (2020) utilized grower pigs and focused on soybean hull inclusion at 7.5 or 15% and unground or ground soybean hulls at 787 μm or 370 μm, respectively.

Nitrogen retention was reduced as soybean hull inclusion increased which conflicts with Lee et al. (2022) who reported higher nitrogen retention rates in high-insoluble fiber diets compared to a low-fiber diets. However, the retention of N as a percent of intake decreased, which is supported by similar findings from Miller et al. (2025). Reduced crude protein digestibility in the current study led to increased fecal N excretion with the addition of soybean hulls consistent with other studies that observed no differences in total N excretion but of that total, a majority was excreted via feces over urine (Zervas and Zijlstra 2002; Shriver et al. 2003; Mpendulo et al. 2018; Lee et al. 2022). However, in disagreement with the observed reduction in urinary N excretion in the present study, Lee et al. (2022) determined that urinary N excretion was not affected by dietary treatment. In diets that contain no dietary fiber, N is partially converted to ammonia in the gastrointestinal tract, which can then be absorbed and excreted in urine as urea (Mosenthin et al. 1992). In diets high in fiber, microbes in the large intestine use fiber as an energy source and ammonia as a nitrogen source for de novo synthesis of proteins (Morgan and Whittemore 1988). Similarly, dietary soluble fiber leads to a decrease in urinary N excretion as microbiota in the large intestine use the N to sustain their growth (Jha and Berrocoso 2016). As a result, the ammonia N may be excreted as microbial protein in the feces instead of being absorbed and excreted in the urine (Morgan and Whittemore 1988; Mosenthin et al. 1992).

The feeding of increasing levels of soybean hulls resulted in decreased ATTD of ash, dry matter, crude protein, and organic matter. Blank et al. (2012) observed reductions in DM, OM, and CP digestibility when using fiber sources such as wheat bran, rapeseed, cassava leaf, and cassava root which all contain greater than 65% insoluble fiber. Similar reductions in digestibility were reported by Lee et al. (2022) who compared high-fiber, high-soluble fiber, and high-insoluble fiber diets to a control diet. Oh et al. (2020) reported reduced nutrient digestibility when replacing corn with 10% soybean hulls. Jaworski and Stein (2017) reported reductions in the digestibility of DM with the addition of 29% soybean hulls compared to a basal diet, a basal diet with added dried distiller’s grains with soluble (DDGS) or a basal diet with added wheat middlings. Renteria-Flores et al. (2008) reported that N digestibility decreased as soybean hulls inclusion increased in diets for grower pigs, with reductions observed at soybean hulls inclusion levels up to 25%. Zervas and Zijlstra (2002) reported similar findings with both soybean hulls and sugar beet pulp.

Soluble fiber (e.g., pectins and gums) is more digestible than insoluble fiber (e.g., cellulose, hemicellulose, and lignin); therefore, fiber source composition can influence digestibility (Noblet and Le Goff 2001). Soluble fiber has a higher WHC than insoluble fiber, which can increase digestibility (Lindberg 2014). Estimation of energy digestibility of fiber rich ingredients are also time dependent. Gao et al. (2023) reported that at least a seven-day adaptation period was required to allow for hindgut microbiota acclimation, while Choi and Kim (2019) reported that a low fiber diet requires a slightly longer adaptation period (6 days compared to 4) compared to a high fiber diet before the collection of feces for digestibility work. In this study, the authors provided a three-day acclimation period before the initial collection and proceeded with two additional collections to account for potential microbial acclimation.

As soybean hull inclusion increased in the diet, digestible and metabolizable energy declined. Dietary concentrations of ME were influenced by soybean hulls inclusion in the current study, with the 40% soybean hulls diet resulting in the lowest ME (2.89 Mcal/kg) compared to the other dietary treatments of 0%, 10% and 20% inclusion, respectively (3.32 Mcal/kg, 3.27 Mcal/kg, and 3.17 Mcal/kg, as-fed). This aligns with Kornegay (1981); who reported that increasing soybean hull inclusion (0 to 30% inclusion) lead to a reduction in diet ME (3.2 Mcal/kg vs 2.91 Mcal/kg). Additionally, energy values increased over time in this study, which was not unexpected. As a pig adapts to a high fiber diet, increases in intestinal mass occur, allowing the pig to capture more energy through fermentation of the fibrous feed stuff (Jha et al. 2019).

To provide context relative to previously reported soybean hull energy values, an approximate ME value for the soybean hull source used in this experiment was derived from a secondary analysis of the observed diet-level response using the regression method (Adeola 2000). Least squares means of diet ME (kcal/kg as-fed) were first expressed as differences relative to the 0% soybean hull diet. These delta ME values were then regressed against soybean hull inclusion using a linear model with the intercept constrained to zero, indicating that diet ME decreased by approximately 9.7 kcal per 1% increase in soybean hull inclusion. Scaling this relationship to the average energy-yielding fraction of the experimental diets (98.2%) and adding the resulting value to the ME of the control diet (3,315 kcal/kg as-fed) yielded an approximate soybean hull ME value of 2,362 kcal/kg as-fed averaged across the 3-week period. This estimate should be interpreted cautiously, as the experimental diets were not formulated as a pure basal substitution series and soybean hulls replaced a mixture of energy-yielding ingredients across treatments; accordingly, this value is presented only to place the magnitude of the observed diet ME response in the context of previously published soybean hull energy values, rather than as a definitive ingredient ME estimate (Noblet et al. 2022).

The NRC (2012) reported the ME value of soybean hulls to be 1.94 Mcal/kg, which is lower than the value in this study reported. Miller et al. (2025) reported a value in a grower study after pigs had been on treatment diets for approximately eight weeks of 2.44 Mcal/kg in a diet containing 22.5% soybean hulls. Differences between the determined ME values of soybean hulls across studies could be explained by differences in soybean hulls dietary inclusion, source, or particle size.

## Conclusion

Feeding increasing levels of soybean hulls up to 40% resulted in a decreased digestibility of DM, CP, and OM, with the most severe impact noted in the diet containing 40% soybean hulls. Reduced N digestibility increased fecal N excretion and decreased urinary N excretion. Total N excretion did not change but the proportion excreted in feces relative to urine was greater with higher dietary levels of soybean hulls. Increasing the inclusion of soybean hulls reduced the ME value of the diet.
